# Review of Techniques for Improvement of Softening Behavior of Age-Hardening Aluminum Alloy Welded Joints

**DOI:** 10.3390/ma14195804

**Published:** 2021-10-04

**Authors:** Jiwen Cheng, Gang Song, Xiaosheng Zhang, Chunbai Liu, Liming Liu

**Affiliations:** 1 Key Laboratory of Liaoning Advanced Welding and Joining Technology, School of Materials Science and Engineering, Dalian University of Technology, Dalian 116024, China; chengjiwen@mail.dlut.edu.cn (J.C.); liulm@dlut.edu.cn (L.L.); 2 China FAW Group Co., Ltd, Manufacturing Engineering & Logistics Department, Changchun 130011, China; zhangxiaosheng@faw.com.cn (X.Z.); liuchunbai@faw.com.cn (C.L.)

**Keywords:** age-hardening aluminum alloys, joint softening, low-heat-input welding, externally assisted cooling during welding, post-weld treatment

## Abstract

The softening phenomenon of age-hardening aluminum alloy-welded joints is severe during conventional fusion welding, which increases the likelihood of stress and strain concentration in the joint during the period of service, significantly reduces the mechanical properties compared to the base metal, and represents an obstacle to the exploration of the potential structural performance. This review paper focuses on an overview of the softening phenomenon. Firstly, the welding softening mechanism and the characteristics of age-hardening aluminum alloys are clarified. Secondly, the current main research methods that can effectively improve joint softening are summarized into three categories: low-heat-input welding, externally assisted cooling during welding, and post-weld treatment. The strengthening mechanism and performance change rule of age-hardening aluminum alloy joints are systematically analyzed. Finally, this paper considers the future development trends of further research on joint softening, and it is expected that interest in this topic will increase.

## 1. Introduction

### 1.1. Background

Age-hardening aluminum alloys are widely used in structural design in modern aerospace, automotive, and other transportation industries due to their high specific strength, good formability and corrosion resistance, which can meet the requirements of light weight under the premise of ensuring safety and reliability [1,2,3]. However, one of the major challenges in the manufacturing process is the need to weld the age-hardening aluminum alloys, which are extremely sensitive to welding heat input due to their precipitation strengthening mechanism [2]. Moreover, the joint efficiency of age-hardening aluminum alloys welded by conventional fusion welding, such as tungsten inert gas (TIG) and metal inert gas (MIG) welding with low energy density and high heat input characteristics, is usually reduced to less than 50% [4]. This performance degradation in the welded joints is mainly due to the softening phenomenon in the fusion zone (FZ) and heat−affected zone (HAZ), which is caused by precipitation dissolution, transformation and coarsening [5]. The significant deterioration in the mechanical properties of welded joints compared with the base metal reduces the structural bearing limit and service life, which is unable to meet the performance requirements and limits the application field of age-hardening aluminum alloys. Therefore, it is necessary to take measures to improve the mechanical properties of age-hardening aluminum alloy-welded joints.

### 1.2. The Classification of Aluminum Alloys

**Figure 1:**
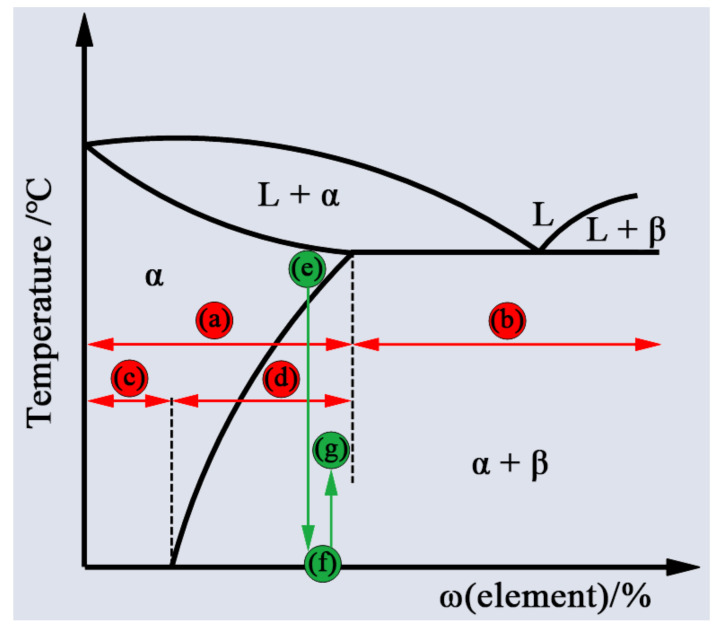
Schematic diagram of the classification of aluminum alloys (red line) and solution, quenching and aging heat treatment process (green line). (**a**) Wrought aluminum alloy; (**b**) cast aluminum alloy; (**c**) non-heat-treatable aluminum alloys; (**d**) heat-treatable aluminum alloys; (**e**) solution heat treatment; (**f**) quenching treatment; (**g**) aging treatment.

According to the strengthening mechanisms of aluminum alloys, they can be divided into two categories: (I) heat-treatable aluminum alloys and (II) non-heat-treatable aluminum alloys [6], as shown in Figure 1. By controlling a sequence of thermal treatment processes involving solution heat treatment and quenching, the aluminum alloys that have the capability of forming a supersaturation solid solution and can be strengthened by the consequent formation of coherent fine precipitates with aging treatment are referred to as “heat-treatable aluminum alloys” [6,7]. In contrast, the aluminum alloys that cannot be strengthened through heat treatment and can be effectively strengthened through work hardening are described as “non-heat-treatable aluminum alloys” [6,8]. Table 1 shows the different classifications of aluminum alloys based on their main alloying elements; from the table, it can be seen that the 1XXX, 3XXX, 4 XXX and 5XXX series alloys are non-heat-treatable aluminum alloys, while the 2XXX, 6XXX, 7XXX and some 8XXX series alloys are heat-treatable aluminum alloys, which is due to the type of master alloying elements, which can generate the effect of precipitate strengthening by the thermal treatment process. The classification of aluminum alloys is commonly followed by a temper designation, which includes F (as fabricated), O (annealed), H (strain hardened), W (solution heat-treated) or T (thermally treated), to provide information about the fabrication process and treatment mode [9,10,11]. Table 2 provides a detailed description of the five basic temper designations. The heat-treatable aluminum alloys under T temper, which are strengthened by the aging process, are referred to as “age-hardening aluminum alloys”. Numerals 1 to 10 following the T indicate the main type of heat treatment, as listed in Table 3, and the second to fifth digit (if present) indicate the stress release or other special treatments [10].

### 1.3. The Strengthening Mechanism of Age-Hardening Aluminum Alloy

Table 4 summarizes the mechanical properties of age-hardening aluminum alloys. It can be seen that the temper and chemical compositions of age-hardening aluminum alloys have a great influence on their mechanical properties. This is due to the different thermally treated processes and chemical compositions, which affect the characteristics of the precipitation phase, such as the type, quantity, shape, size and distribution [7]. The characteristics of the precipitation phase can affect the strengthening effect of age-hardening aluminum alloys and thus influence their mechanical properties.

**Figure 2:**
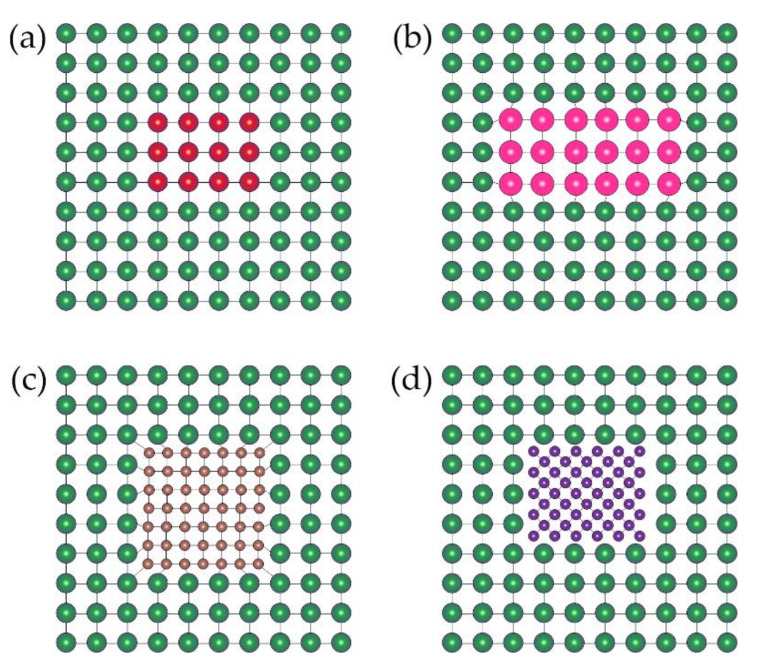
Schematic diagram of precipitation sequence of aluminum alloys: (**a**) Guinier-Preston zone; (**b**) coherent metastable phase; (**c**) semi-coherent metastable phase; (**d**) non-coherent equilibrium phase.

**Figure 3:**
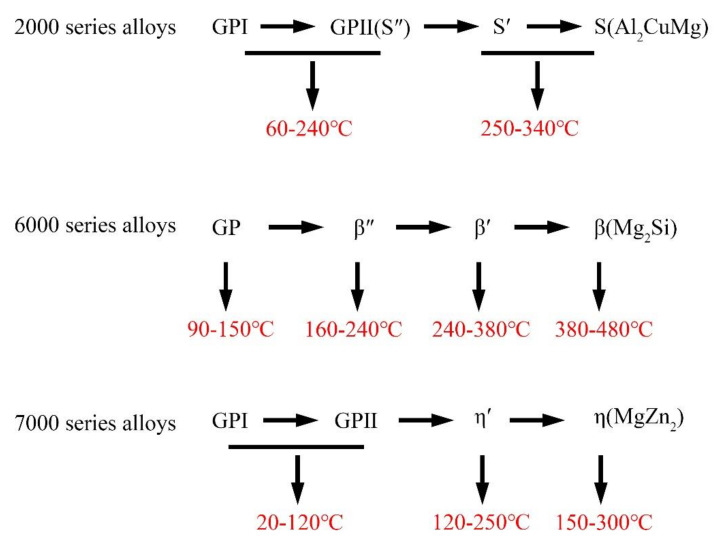
The temperature range of different strengthening phases precipitated (according to [34,35,36]).

At present, the generally accepted precipitation sequence of the strengthening phase in age-hardening aluminum alloy is supersaturated solid solution→GP zone→coherent precipitate→semi-coherent precipitate→non-coherent precipitate, as shown in Figure 2. It can be noted from Figure 3 that when aging in a lower temperature range, the precipitates in aluminum alloy are mainly in the GP zone. During aging in a higher temperature range, the GP zone gradually changes to coherent and semi-coherent precipitates. With a further increase in aging temperature, the precipitates of aluminum alloy are mainly stable, non-coherent precipitates, and the non-coherent precipitates gradually coarsen with the increase in time.

The age-hardening aluminum alloy in the peak-aged state has high strength owing to a large number of precipitates that are very fine and coherent, and contributes to the effective resistance to dislocation movement and can cause an intense strengthening effect [13,14]. However, when the coherent, metastable precipitates gradually convert to the semi-coherent metastable phase or non-coherent equilibrium phases and are coarsened quickly, the strengthening effect dramatically declined, and the age-hardening aluminum alloy is then in an over-aged state [7].

**Table 4:** Summary of the mechanical properties of age-hardening aluminum alloys (yield strength (YS), ultimate tensile strength (UTS), elongation (El)).

Materials	Temper	Elements													YS (MPa)	UTS (MPa)	El (%)	Ref.
		Cu	Mg	Li	Mn	Zn	Fe	Si	Zr	Cr	Ti	Ag	V	Al				
2014	T651	4.8	0.54		0.38	0.25	0.7	0.97						balance	410	469	9.14	[15]
2024	T3	4.42	1.56		0.61	0.13								balance	468	506	16.8	[16]
2024	T3	4.9	1.28		0.629			0.43		0.012				balance	317	448	14	[17]
2024	T351	4.5	1.4		0.6	0.03	0.17	0.05		0.01	0.02			balance	360	470	20.3	[18]
2050	T84	3.6	0.38	0.98	0.32	0.12	0.03		0.08		0.03	0.48		balance	465.9	553.3	15.13	[19]
2195	T8	4.0	0.44	1.0			0.05		0.11			0.4		balance	571.5	609.7	7.4	[20]
2195	T8	3.99	0.38	1.09			0.17		0.1			0.42		balance	580	615	9	[21]
2219	T6	6.48			0.32	0.04	0.23	0.49	0.2		0.06		0.08	balance	345	416	15	[22]
2519	T87	5.71			0.27		0.1	0.04			0.02		0.05	balance	427	452	11.2	[23]
6005	T6	0.25	0.55		0.45		0.19	0.55		0.13	0.08			balance	272	285	8.4	[24]
6061	T6	0.21	0.87		0.056			0.61		0.104				balance	259	287	11	[17]
6061	T913	0.2	0.89		0.04		0.37	0.64						balance	455	460	10	[25]
6063	T6	0.002	0.525		0.002	0.002	0.09	0.412		0.001	0.014			balance	172	230	13.5	[26]
6082	T6	0.08	0.78		0.48	0.04	0.39	0.95		0.03	0.05			balance	283	324.8	12.2	[27]
6101	T6	0.05	0.65		0.03		0.5	0.5						balance	195	220	15	[28]
6351	T6	0.1	0.8		0.7		0.6	0.95						balance	285	310	14	[28]
7039	T6	0.05	2.37		0.68	4.69	0.69	0.31						balance	328	414	15.1	[29]
7050	T76	2.1	2.2		0.0079	6.187	0.06	0.03	0.11	0.0048	0.02			balance	520	559	10	[30]
7075	T6	1.5	2.5		0.04	5.6	0.3	0.08						balance	404	508	15	[31]
7075	T6	1.51	2.38		0.02	5.41	0.25	0.07		0.19	0.02			balance	415.5	453	13.9	[22]
7075	T651	2.0	2.9		0.3	6.1	0.5	0.4		0.28	0.2			balance	510	563	16	[32]
7075	T651	1.7	2.4		0.04	5.8	0.19	0.05		0.2	0.03			balance	476	555	11.4	[18]
7N01S	T5	0.011	1.34		0.317	4.19	0.1	0.046	0.122	0.233	0.043			balance	327	393	15.5	[33]

### 1.4. The Welding Softening Characteristics of Age-Hardening Aluminum Alloy

The mechanical properties of age-hardening aluminum alloys highly depend on the strengthening phase, which precipitates at a specific temperature range [34,35,36]. Therefore, during the conventional fusion welding process, the performance of age-hardening aluminum alloy-welded joints that include FZ and HAZ is significantly different from the base metal (BM) owing to the strengthening phase dissolution, transformation and coarsening. Under the effect of the welding thermal cycle, the strengthening phase over-aging results in joint softening, which contributes to low joint efficiency. Three factors affect joint efficiency:

(1) The characteristics of the precipitation phase in BMs

The characteristics of the precipitation phase in BMs, which is determined by the temper and chemical composition, also have a great influence on the joint efficiency. Compared to peak-aged aluminum alloys, the underaged or overaged aluminum alloys have lower mechanical properties and their precipitation is less sensitive to the welding thermal cycle. Therefore, the joint efficiency of peak-aged aluminum alloys is lower than that of under-aged or over-aged aluminum alloys.

(2) The thickness of welding plates

For the same temper and chemical compositions of age-hardening aluminum alloys, thicker plates need a larger welding heat input than thinner plates to achieve sufficient penetration [37,38], which causes the joint efficiency of thicker plates to be lower due to the more serious softening in FZ and HAZ.

(3) Welding technique and process

The welding method with low energy density and the welding process with large heat input would significantly reduce the joint efficiency due to more serious softening in FZ and HAZ.

**Figure 4:**
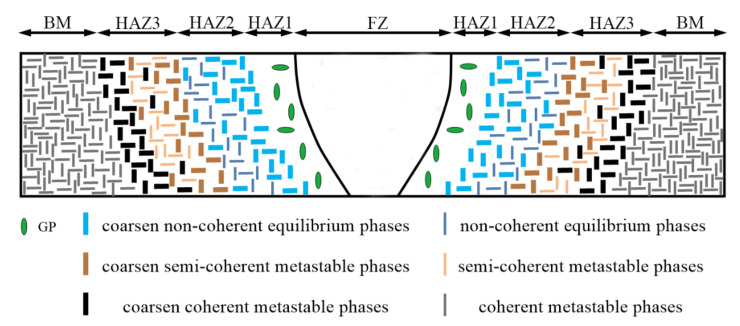
A schematic diagram of the evolution of the strengthening phases for a cross-section of a welded joint.

For aged-peak aluminum alloys, joints welded by conventional fusion welding can roughly be divided into five areas, namely FZ, HAZ1, HAZ2, HAZ3 and BM, that reflect the effect of different temperatures on the strengthening phases, as shown in Figure 4. The FZ and nearby HAZ1 are exposed to temperatures that exceed the solvus line during welding, which results in a large number of coherent strengthening phases dissolving and forming a supersaturated solid solution and GP region during subsequent cooling [39]. The peak temperature of the severe softening HAZ2 zone is just below the solvus line, so coherent strengthening phases disappear and a great amount of non-coherent equilibrium phases are precipitated and coarsened, which leads to the mechanical properties of this zone dramatically declining [39]. The slight softening zone is referred to as HAZ3 near to the BM; the the peak temperature range of this zone is lower than that of HAZ2 and can only cause some coherent strengthening phases to be coarsened or transformed into semi-coherent phases [40].

It is obvious from the above-mentioned discussion that the larger the welding heat input, the greater the degree and range of age-hardening aluminum alloy joint softening. Moreover, in contrast to other welding defects, such as porosity and cracks, that can be eliminated by improving the welding process, softening is inevitable. Hence, improving the effect of welding heat on the microstructure and properties of age-hardening aluminum alloy joints has increasingly become the focus of researchers.

### 1.5. Research Status and Progress

**Figure 5:**
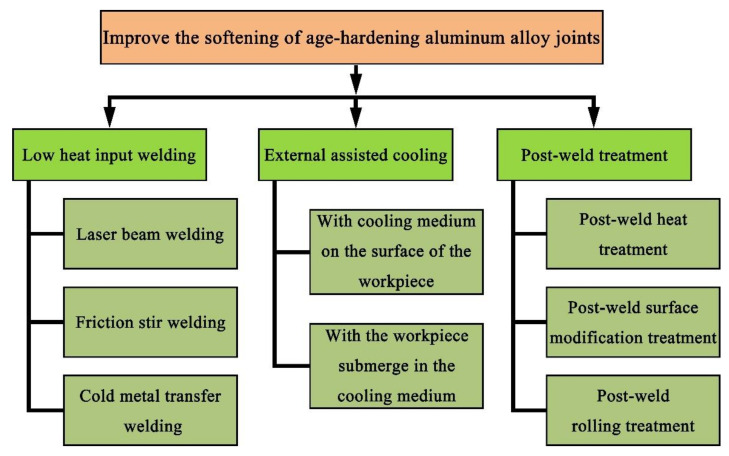
Outline of research based on reducing joint softening.

According to the characteristics of softening in age-hardening aluminum alloy joints, researchers have adopted various effective technologies and processes to reduce the joint softening and improve the performance of welding joints, which can be summarized into three categories, as shown in Figure 5.

(1) Low-heat-input welding: such as laser beam welding (LBW), friction stir welding (FSW), and cold metal transfer (CMT) welding. To improve the mechanical properties of age-hardening aluminum alloy joints welded by the low heat input technique, researchers have focused on how to eliminate welding defects [41,42,43,44], optimize welding parameters [45,46,47] and innovate the technique [48,49,50,51,52,53].
(2) Externally assisted cooling: involving spraying a cooling medium onto the surface of the workpiece and submerging the workpiece in the cooling medium. To reduce the effect of the welding thermal cycle on strengthening precipitates, researchers have sought to control the peak temperature range and enhance the cooling rate during welding [54,55].
(3) Post-weld treatment: including post-weld heat treatment, post-weld surface modification treatment and post-weld rolling treatment. To further improve the mechanical properties of age-hardening aluminum alloy joints, researchers have sought to strengthen the softening zones through post-weld treatment, causing over-aged strengthening phases to reprecipitate or introducing dislocation strengthening [56,57,58].

## 2. Low-Heat-Input Welding

The precipitation strengthening mechanism of age-hardening aluminum alloys is easily affected by the welding heat input. The high heat input undoubtedly results in extensive over-aging of strengthening phases and increases the degree and range of joint softening, which seriously affects the mechanical properties of welded joints. Therefore, in recent years, welding research on age-hardening aluminum alloys has mainly focused on welding methods with low heat input characteristics, such as LBW, FSW and CMT. Although the mechanical properties of joints produced with low-heat-input welding methods have been improved, there are still some welding problems. Based on this, researchers have made great efforts to solve these problems, so that the mechanical properties of the joints can be further improved and the application scope of the low-heat-input welding method can be expanded.

### 2.1. LBW

For age-hardening aluminum alloys using conventional fusion welding with a low energy density, excessive heat input often causes the weak position of the joint to be located in the FZ or HAZ; thus, the performance of the welded joint severely declines, and the joint efficiency can be reduced to less than 50% [4]. However, LBW with a concentrated heat source is able to achieve deep penetration and high welding speeds, thereby leading to a low heat input during the welding process. Subsequently, it results in the formation of a narrow FZ and HAZ, and therefore, the range and degree of performance decline caused by thermal degradation are small [59], and the joint efficiency of LBW is greater than that of conventional fusion welding, as shown in Table 5. Table 5 shows that the weak position of the autogenous LBW joint is in the FZ. It is mainly caused by the loss of the strengthening phases in the weld metal, as well as the formation of metallurgical problems and welding defects [44,60]. The characteristics of precipitated phase [44,60], metallurgical microstructure [42], porosity [41,42] and cracking in weld play an important role in determining the performance of the joint. Adding an appropriate filler material can reduce the welding defects and increase the ultimate tensile strength by approximately 7% when compared to autogenously laser-welded specimens [61]. Consequently, many researchers have made efforts to improve the performance of the FZ of LBW by adjusting the chemical composition, reducing cracking susceptibility and controlling the solidification microstructure.

#### 2.1.1. Adjusting the Chemical Composition in FZ

**Figure 6:**
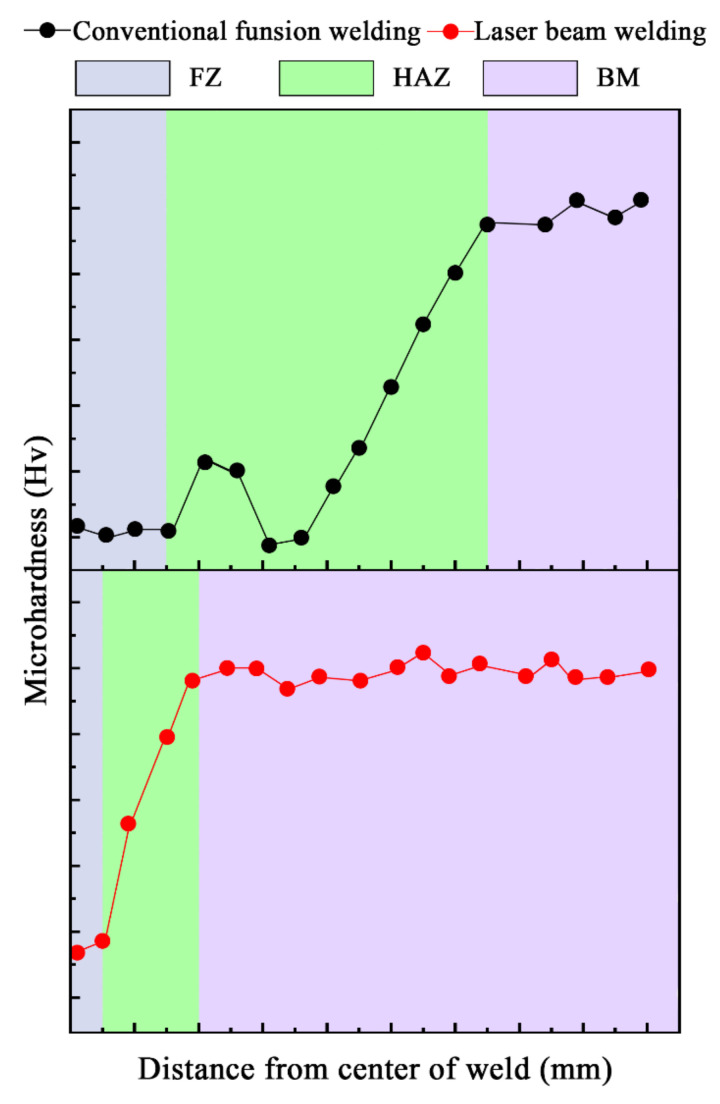
Typical microhardness distribution of AA7XXX series with T6 temper joint cross-section by conventional fusion welding and LBW (according to [70,73]).

**Figure 7:**
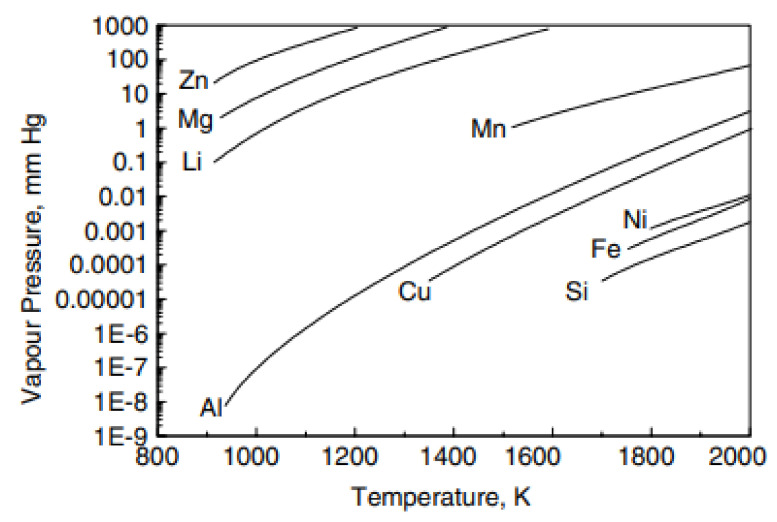
The relationship between equilibrium vapor pressure and temperature for elements [73,78].

Compared to conventional fusion welding, Figure 6 shows the typical microhardness distribution of the AA7XXX series under a T6 temper joint cross-section welded by autogenous LBW. It can be seen that the width of the FZ and HAZ of the LBW joint is much narrower than that of conventional fusion welding joints. The microhardness in the HAZ of LBW is higher than that of conventional fusion welding joints. The lowest microhardness of the LBW joint is in the FZ. The FZ softening caused by the loss of precipitating strengthening phases is due to the evaporation of magnesium and zinc alloying elements [71]; in addition, the welding temperature exceeds the solvus line, resulting in the formation of a supersaturated solid solution [39] and non-equilibrium solidification, leading to the segregation of alloy elements [62,72,73]. It can be seen from Figure 7 that Mg and Zn as main elements are added to age-hardening aluminum alloys to promote precipitate strengthening as they possess lower boiling points (Mg (1091 °C) and Zn (917 °C)) and much higher vapor pressure than other elements; therefore, they are excessively vaporized during welding [74]. Ola et al. [75] suggested that LBW with a suitable filling wire could compensate for the loss of volatile alloying elements. In addition, Zhang et al. [76] reported that a small quantity of Zr and Er elements added to the filling wire could refine the as-cast microstructure in the FZ and promote the formation of fine and uniform equiaxed grains; therefore, the dendritic segregation could be eliminated [76,77].

#### 2.1.2. Reducing the Cracking Susceptibility

**Figure 8:**
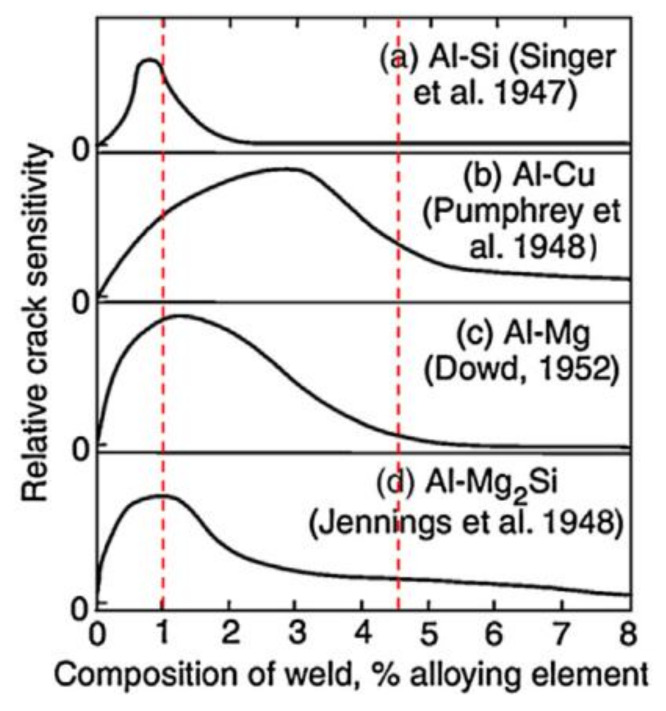
The effect of different elements on aluminum crack sensitivity [62]. (**a**) Si; (**b**) Cu; (**c**) Mg; (**d**) Mg2Si.

Compared to pure aluminum, the addition of alloying elements such as silicon, copper, magnesium and zinc to aluminum to generate the effect of precipitate strengthening by the thermal treatment process can increase the crack susceptibility in the FZ during autogenous laser welding [62]. The high content of alloying elements results in a wide solidification temperature range and the presence of eutectic constituents with a lower melting point than the base metal along the grain boundary in the FZ [59]. The properties of aluminum alloys, such as the high coefficient of thermal expansion and large solidification shrinkage, result in high stress and strain during welding, which may cause the low melting liquid film of eutectics to separate along the grain boundary, i.e., crack [62]. As shown in Figure 8, different alloying elements and contents have different effects on the crack sensitivity of aluminum; for example, for an aluminum alloy containing 1 wt.% Mg2Si, the crack tendency is maximum. Therefore, the alloying elements can not only increase the strength by precipitation strengthening but also affect the likelihood of welding cracks. Selecting a suitable filling material and adjusting the fusion ratio can control the composition of the weld and reduce the susceptibility to cracks. The welding filler materials of AA2XXX and AA6XXX aluminum alloy generally contain excessive silicon or magnesium elements, such as AlSi5 [62], AlSi7Mg [79], AlSi12 [80]. On the one hand, the welding filling materials can dilute and reduce the content of Mg2Si in the molten pool and cause the peak value of the Al-Mg, Al-Mg2Si solidification crack curve to be outside of the sensitive range. On the other hand, the addition of silicon reduces the solidification temperature and thus reduces the cracking tendency. The welding filler materials of AA7XXX aluminum alloy generally contain excessive magnesium [81], such as ER5183, ER5356, ER556, which makes that the weld composition avoids the Mg content range with the worst crack resistance.

#### 2.1.3. Controlling the Solidification Microstructure

**Figure 9:**
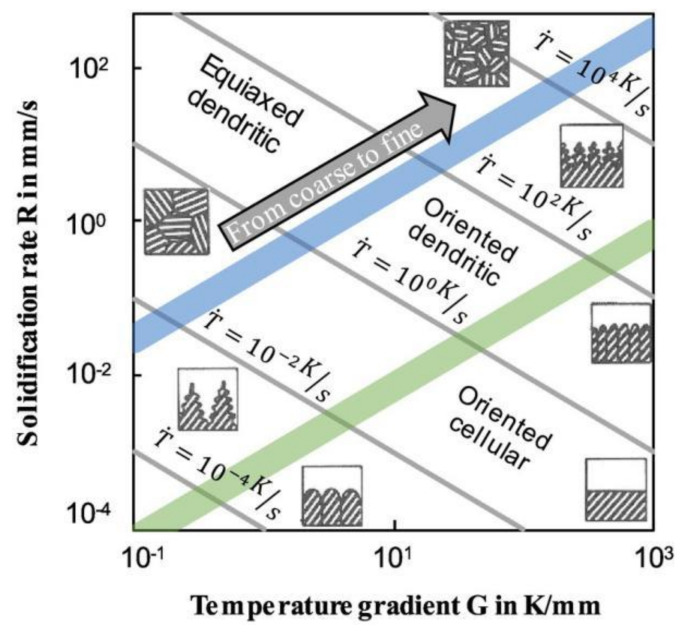
Solidification grain microstructure map [82,83].

For autogenous laser welding, the FZ contains a large number of cellular dendrites; only the center of the FZ is slightly equiaxed. The grain microstructure in the FZ is correlated with the solidification rate and temperature gradient. The solidification grain microstructure map created by Kurz and Fischer is presented in Figure 9.

During the welding process, an equiaxed crystal structure can easily form with a high solidification rate and low-temperature gradient, and cellular dendritic grains can easily form with a low solidification rate and high-temperature gradient. The local solidification rate is as expressed in the following equation [84]:

(1) R=Vcosα

where *R* is the solidification rate, V is the welding speed, and α is the angle between the welding direction and the normal surface of the solidification front, which is determined by the weld shape.

**Figure 10:**
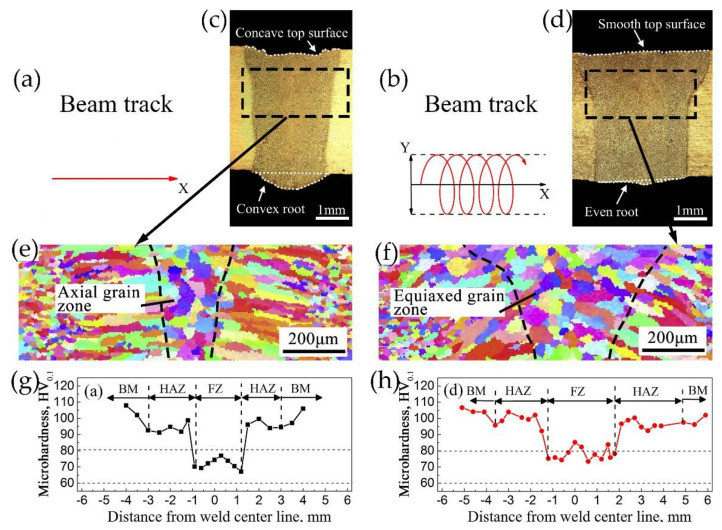
Effect of beam oscillation on grain microstructure and microhardness in the FZ [67]. (**a**,**b**) correspond to the oscillating pattern without oscillation and with circular oscillation, respectively; (**c**,**d**) correspond to the cross-section morphologies without oscillation and with circular oscillation, respectively; (**e**,**f**) correspond to the inverse pole figure (IPF) images of the weld without oscillation and with circular oscillation, respectively; (**g**,**h**) correspond to the microhardness profile across the weld cross-section without oscillation and with circular oscillation, respectively.

Laser welding with beam oscillation can adjust the solidification rate by controlling the weld shape, and thus improve the grain microstructure in the FZ. As shown in Figure 10, LBW with a circular beam oscillation process leads to a larger number of equiaxed grains in the FZ, which improves the microhardness. Hence, Hagenlocher et al. [83] reported that LBW with circular beam oscillation could effectively improve the weld strength and reduce the risk of hot cracking.

#### 2.1.4. Innovations in Laser Welding of Aluminum Alloys

When laser welding thick aluminum alloy structures, the keyhole-welding mode requiring a high-power laser source should be adopted to obtain high penetration depths. However, some critical issues usually arise during the welding process: (I) high energy consumption—the energy density (ratio of the laser power to the beam spot area) for iron-based materials in the keyhole-welding mode is approximately 10^6^ W/cm^2^, while the value for aluminum alloys is at least 1.5 × 10^6^ W/cm^2^ and even 2 × 10^6^ W/cm^2^, because aluminum alloys possess high laser radiation reflection and thermal conductivity [85,86]; (II) keyhole-induced porosity—the low-viscosity aluminum alloys cause the keyhole to become unstable and easily collapse, resulting in the entrapment of the gas bubbles during the cooling, which dramatically reduces the mechanical properties of the joint [87,88]; (III) low gap bridging ability and high requirement in positioning—laser welding with a high welding speedand a small focusing point [89,90,91].

**Figure 11:**
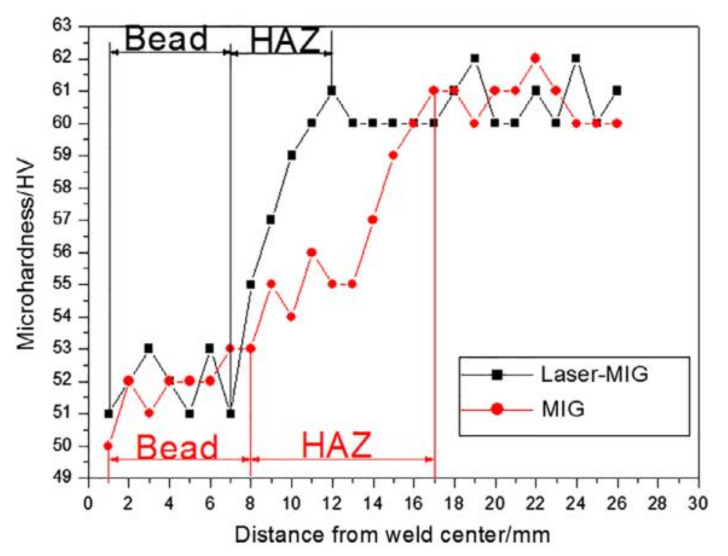
Microhardness of joints welded by hybrid laser–MIG welding [95].

To overcome the shortcomings of laser welding thick aluminum alloys, researchers suggest using the hybrid laser–MIG welding method, which was developed by Steen and Eboo in 1979 [92]. Hybrid welding combines the advantages of laser welding and MIG welding, resulting in an improvement in energy utilization, an improvement in keyhole stability and an increase in gap bridging ability [48,49]. Compared to MIG welding, hybrid laser–MIG has a lower heat input under the same penetration, and improves the joint efficiency, as listed in Table 6. As shown in Figure 11, hybrid laser–MIG can reduce the softening range and softening degree in the HAZ of age-hardening aluminum alloys, and improve the tensile strength of welded joints. Although the degradation of properties in the FZ and HAZ still occurs in joints of thick aluminum alloys produced by hybrid laser–MIG welding, the porosity, as the most serious defect [93], seriously impairs the engineering application of the joint. Based on this, researchers have proposed many effective approaches to reduce the welding porosity, such as optimizing the shielding gas environment, applying a magnetic or ultrasound field during the welding process and optimizing the welding process.

(1) Optimizing shielding gas environment

Cai et al. [99] found that a shielding gas could influence the stability of the welding process; helium could improve the stability of keyholes and suppress the weld porosity defect. A similar conclusion was reported by Ahn et al. [100]; the authors pointed out that helium with high ionization potential reduced the plume effect, which minimized porosity formation. Moreover, the high heat generated in helium-shielded welding resulted in a high weld pool temperature and long solidification time, which helped the bubbles to escape from the molten pool.

(2) Applying a magnetic or ultrasound field during the welding process

**Figure 12:**
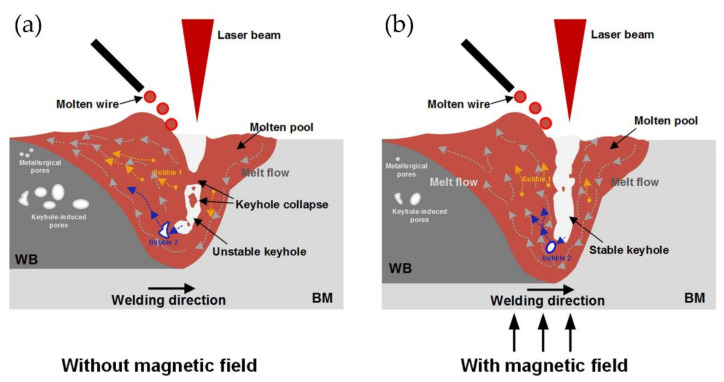
The schematic diagram illustrates the porosity formation and evolution under laser-MIG hybrid welding with and without a magnetic field [101]. (**a**) without magnetic field; (**b**) with magnetic field.

Liu et al. [101] investigated the effect of a magnetic field on porosity in hybrid laser–MIG welding aluminum alloys. As shown in Figure 12, the magnetic field increased the stability of keyholes, and thus decreased the laser-induced bubbles. Moreover, the magnetic field had a stirring effect on the molten pool, which significantly changed the direction of the melt flow, obviously improved the convection intensity of melts and helpfully provided more channels for bubbles to escape from the molten pool. Liu et al. [102] introduced ultrasonic vibration as external energy into the molten pool and found that the cavitation effect of the ultrasonic vibration could allow the bubbles to rapidly escape from the molten pool, thus reducing porosity in joints.

(3) Optimizing welding process

Huang et al. [103,104] reported that, compared to arc-leading hybrid welding, the alloys produced with the laser-leading mode of welding has less porosity owing to a more stable molten pool and keyhole. Leo et al. [105] claimed that the laser power-to arc power ratio has a significant effect on the weld porosity, and the porosity increased with laser power, which was due to the vaporization of the magnesium alloying elements.

### 2.2. FSW

**Figure 13:**
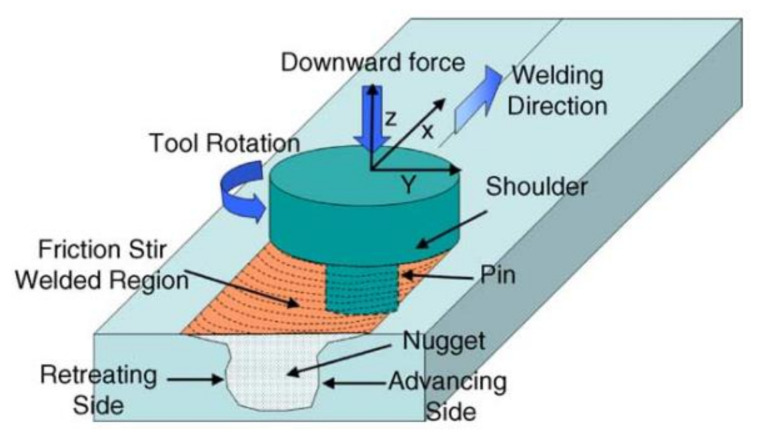
Schematic diagram of friction stir welding [106].

In the FSW process, due to the friction heat between the tool shoulder and the workpiece surface, the material enters into a plastic softening state. Under the effect of the rotating pressure of the pin, the softened material undergoes plastic flow, fills the cavity generated by the moving of the tool and forms the weld [106,107,108], as shown in Figure 13. Because the heat generated by the welding process does not melt the base metal, a range of defects that are produced in the fusion welding process, such as cracks and porosity, can be avoided [109,110,111,112]. Therefore, FSW is widely used to weld non-ferrous metal, especially for age-hardening aluminum alloys. Table 7 shows the tensile properties of an age-hardening aluminum alloy butt joint welded by conventional FSW.

#### 2.2.1. Optimization of Welding Process Parameters

Although the heat input of FSW is lower than that of conventional fusion welding, the microstructure morphology and properties of welded joints are still changed under the influence of the welding thermal cycle [138,139,140,141]. For age-hardening aluminum alloys, the type, size, morphology, distribution and density of strengthening phases across the NZ, TMAZ and HAZ mainly dominate the joint strength [140]. Therefore, it is necessary to employ suitable welding process parameters to reduce the influence of the welding thermal cycle on the strengthening phases and properties of joints [45].

The process parameters that can affect the joint performance of FSW aluminum alloys include the shape of the pin and shoulder [142], the tool tilt angle [143], the rotation speed [45], the welding speed [144] and so on. However, the heat input per unit can be calculated by the following equation [46,145,146]:

(2) $$Q=\frac{4}{3}\pi^{2}{\alpha \mu \mathrm{PR}}^{3}\frac{\omega }{v}$$

where Q is the welding heat input per unit, α is the welding heat input efficiency, μ is the friction coefficient, P is the pressure, R is the radius of the shoulder, ω is the rotation speed, and v is the welding speed.

As the welding situation is fixed, only the rotating speed and welding speed directly determine the welding heat input during the friction stir welding process [147,148], and they affect the grain size and characteristics of strengthening phases in NZ, TMAZ and HAZ. Based on this, many scholars have studied the effect of the relationship between rotating speed and welding speed on the microstructure and properties of the joints [46,47,140].

**Figure 14:**
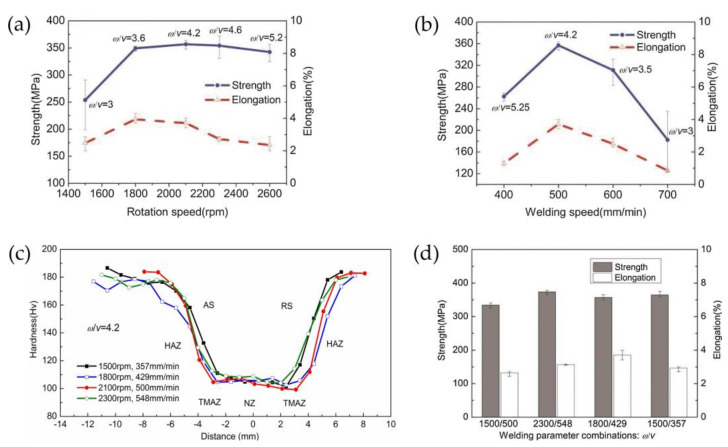
The effect of welding parameters on mechanical properties of friction stir welding [140]. (**a**) The effect of rotation speed on mechanical properties of joints; (**b**) the effect of welding speed on mechanical properties of joints; (**c**) micro-hardness profiles for cross-sections of joints under different combinations of rotation speed and welding speed (ω/v = 4.2); (**d**) the effect of welding parameters on tensile strength and engineering elongation of the joints.

Most studies have shown that [46,140], with an increase in the ω/v value, the joint strength and elongation increase first and then decrease, and only when the value of ω/v is moderate can the joint obtain the best performance. Moreover, researchers reported that for FSW with 2.5 mm 2195-T8 [140], as shown in Figure 14, despite the differences in the performance of joints with different ω/v values, if the different welding speeds and rotation speeds are combined with the same ω/v, the performance of joints was still different due to the different width of the HAZ.

Researchers found that when the welding speed is too slow, the plastic metal accumulates on the backward side of the stirring pin, which leads to the formation of voids in the forward side due to the lack of sufficient metal filling [144]. However, if the welding speed is too fast or the rotation speed is too slow, the welding heat input cannot promote the sufficient plastic flow of the metal, and voids also appear in the weld [140]. This indicates that the mechanical properties of the joints are not only determined by the joint softening but also depended on the welding defects [140]. Hence, to improve the mechanical properties of the joints, it is necessary to select not only a moderate value of ω/v but also a moderate welding speed and stirring needle rotation speed.

#### 2.2.2. Innovations in FSW of Aluminum Alloys

It is well known that reducing the welding heat input can effectively decrease the softening of age-hardening aluminum alloy joints and improve the strength of the joint. However, many studies reported that the low heat input during the FSW can decrease the plastic flow capacity of the material and thus introduce welding defects, such as kissing bonds, voids, tunnels and “S” lines, which dramatically reduce the strength of the joint [50,149]. Hence, to achieve high-strength FSW joints, it is necessary to solve the contradiction between joint softening by high heat input and welding defects by low heat input.

Blaha and Langenecker found that the use of ultrasonic vibration could reduce the yield stress and promote the plastic deformation of the metals [150], which was similar to heat softening. While the heat energy is uniformly absorbed by the metals, the ultrasonic energy can only be absorbed by the crystal defects in the metal [151]. Moreover, the plastic deformation of metals only depends on dislocation movement. To generate the same plastic deformation, the ultrasonic energy needed to promote dislocation movement is far lower than the heat energy. Therefore, ultrasonic-assisted FSW can be used to enhance the plastic flow ability of age-hardening aluminum alloys, and the temperature rise compared to conventional FSW can be negligible. High-strength ultrasonic-assisted FSW joints can be obtained under low-heat-input conditions.

In addition, Hu et al. [50,51,152] systematically studied the ultrasonic-assisted FSW of AA2219-T6 aluminum alloy. Compared to FSW joints, they found that ultrasonic-assisted FSW joints had superior mechanical properties, which could be attributed to ultrasonic-enhanced precipitation strengthening. The excess vacancies promoted the nucleation and precipitation of strengthening phases.

### 2.3. CMT Welding

**Figure 15:**
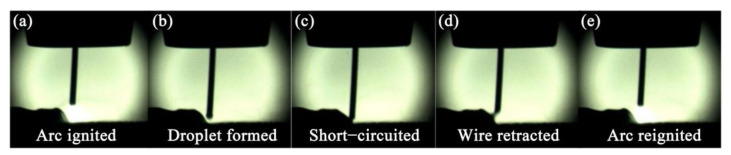
Phases of CMT process [153]. (**a**) arc ignited; (**b**) droplet formed; (**c**) short circuited; (**d**) wire retracted; (**e**) arc reignited.

During the traditional MIG welding process, when the liquid bridge achieves contact with the molten pool, the electromagnetic shrinkage force acting on the liquid bridge is increased by instantly increasing the short-circuit current, which causes the successful metal droplet transition to the molten pool. The increase in short-circuit current will undoubtedly increase the welding heat input. The CMT technique was developed on the basis of the traditional MIG welding technique. However, when the liquid bridge achieves contact with the liquid molten pool, the CMT welding machine control system reduces the shorting-circuit current to almost zero, and the metal droplet transition to the molten pool is aided by the back-drawing force exerted by the wire feeding system [4], as shown in Figure 15.

#### 2.3.1. The Characteristics of CMT Joints

Comparing CMT with other types of fusion welding, many researchers have confirmed that CMT has “colder” characteristics. The characteristics of CMT, with low heat input and less spatter, are highly suitable for welding age-hardening aluminum alloys that are easily affected by the welding temperature and cause serious deterioration in properties [4,154,155,156,157,158]. Cornacchia et al. [159] comparatively studied CMT and MIG welding for a 3 mm EN AW-6005A-T6 with ER4043 filler wire, and the joint efficiency for CMT and MIG welding was 63.6% and 58.7%, respectively. Results indicated that CMT could cause the joint to have better tensile strength compared to MIG welding. Elrefaey [154] carried out the CMT technique for welding 2 mm AA7075-T6 with ER5356 as a filler material, and the joint efficiency was approximately 60%, which was comparable to the FSW and LBW processes, but the fracture position remained in the FZ. Table 8 shows the tensile properties of an age-hardening aluminum alloy butt joint welded by conventional CMT.

#### 2.3.2. Innovations in CMT of Aluminum Alloys

**Figure 16:**
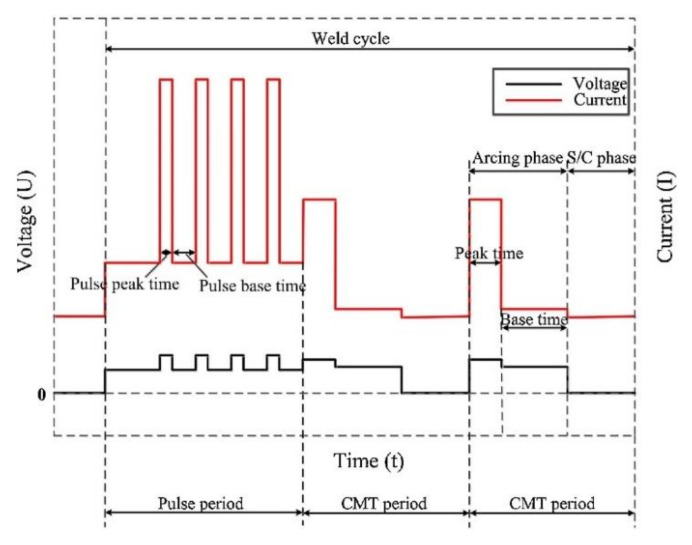
Schematic drawing of a typical CMT + P weld electrical signal [164].

Although the conventional CMT technique is suitable for welding age-hardening aluminum alloys due to its low heat input, the thickness of base metals is limited to no greater than 3 mm, also due to the low energy input [52,164]. If the welding current is increased to weld thick plates, the joint performance will deteriorate. Gandhi et al. [165] carried out CMT double-side welding with a 6 mm 7075 with ER5356 filler wire, and the tensile strength was only 31.8%, corresponding to the base metal. However, age-hardening aluminum alloys used in the structural components of aircraft, high-speed trains, automotives and other fields are not only thin sheets but also thick sheets, which restrict the engineering applications of the conventional CMT technique. To expand the application of CMT, researchers usually adopt two methods.

(I) Hybrid CMT and another type of welding: Liang et al. [52] adopted TIG–CMT hybrid welding to join 4 mm 6061-T6. During the welding process, the TIG arc and the CMT arc did not interact; thus, adding the TIG arc did not cause the droplet transfer to be unstable. However, adding the TIG could increase the welding heat input and achieve deep penetration. To avoid serious softening in the HAZ, a low TIG current should be chosen. The highest tensile strength of the joint was approximately 54%, corresponding to the strength of the base metal. Han et al. [53] comparatively studied a 6 mm 6082-T6 aluminum alloy butt joint welded by laser-cold metal transfer hybrid welding and laser-pulsed metal inert gas hybrid welding, and the results showed that the softening degree and range of HAZ with laser-cold metal transfer hybrid welding were smaller than those achieved with laser-pulsed metal inert gas hybrid welding. In addition, the joint efficiency of laser-cold metal transfer hybrid welding could reach 84.7%, which was higher than that of the laser-pulsed metal inert gas hybrid welding, which was 82.4%. Hence, we can conclude that hybrid CMT and high-energy beam welding can obtain higher joint performance than hybrid CMT and arc welding with low energy density. Hybrid CMT and high-energy beam welding can have lower heat input than hybrid MIG and high energy beam welding. The same conclusion has been reported by Zhang et al. [166].
(II) Control arc mode: Pang et al. [164] adopted the CMT + P technique to weld 4 mm 6061-T6. The CMT + P arc mode combined the advantages of the pulse arc mode and conventional CMT arc mode, as shown in Figure 16. The high pulse current provided more heat input during welding and the low short-circuit current guaranteed droplet transfer stability. The pulse stages and the conventional CMT stages could be adjusted; thus, the heat input of CMT + P was adjustable, which means that this technique could be used to weld thick sheets. Li et al. [167] studied the mechanical properties of 6 mm 6061-T6/7N01-T4 dissimilar aluminum alloy joints welded using the CMT + P technique and found that when increasing the CMT/P, which was the ratio of the number of CMT stages to P stages, the heat input decreased. The lower heat input caused the weld joints to present a lack of fusion or other defects. A higher heat input caused the performance of the joint to significantly degrade due to the over-aging of the strengthening phase in the HAZ. At the optimum heat input, the strength of the joint was approximately 60%, corresponding to the strength of the 6061-T6 base metal.

## 3. Externally Assisted Cooling Technique

The strengthening precipitates of age-hardening aluminum alloys are vulnerable to the peak temperature range and high-temperature residence time during welding. Therefore, the externally assisted cooling techniques, which reduce the peak temperature range and enhance the cooling rate during welding, can improve the mechanical properties of age-hardening aluminum alloy joints by controlling the effect of the welding thermal cycle on strengthening precipitates.

### 3.1. FSW with Externally Assisted Cooling

FSW is a solid-state joining technique; although the temperature during welding does not cause the age-hardening aluminum alloys to melt, it is high enough to cause the dissolution and coarsening of strengthening precipitates in the joint [21], forming a softening region with degraded mechanical properties. Therefore, in recent years, researchers have sought to narrow the softening zone and reduce the softening degree of FSW joints.

**Figure 17:**
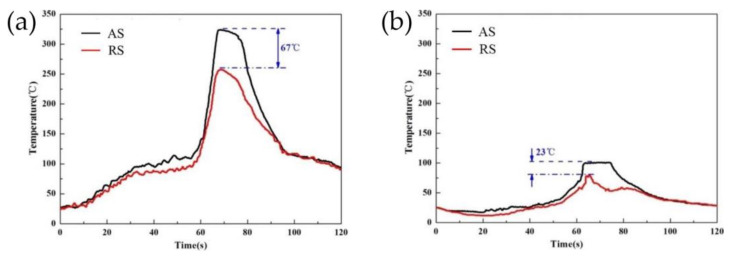
(**a**,**b**) show the thermal curves of joints under air cooling conditions and externally assisted cooling conditions, respectively [55].

Figure 17 shows the welding thermal cycle of FSW under air cooling conditions and externally assisted cooling conditions at a distance of 1 mm from the weld boundary. The AS and RS represent the advancing side of the joint and the retreating side of the joint, respectively. Under air cooling conditions, at the beginning of welding, the temperature of the test point started to rise slowly. When the pin tool was close to the test point, it rose rapidly to 330 °C and then showed a slow cooling rate. However, under externally assisted cooling conditions, the temperature of the test point did not increase until the tool was reached, and the peak temperature could only reach 100 °C After the pin tool moved away, with the strong cooling effect of the cooling medium on the workpiece, the temperature of the test point began to suddenly decline. The same experimental result has also been presented by other researchers [54]. The FSW with externally assisted cooling techniques can effectively lower the peak temperature range and shorten the high-temperature dwelling time of the joint, and the dissolution, transformation and coarsening of strengthening precipitates in the joint are directly associated with the welding thermal cycle, so the externally assisted cooling techniques can restrict the widening of the HAZ and control the degree of softening in the HAZ, which finally results in further improvements in the mechanical properties of FSW joints, as listed in Table 9.

**Figure 18:**
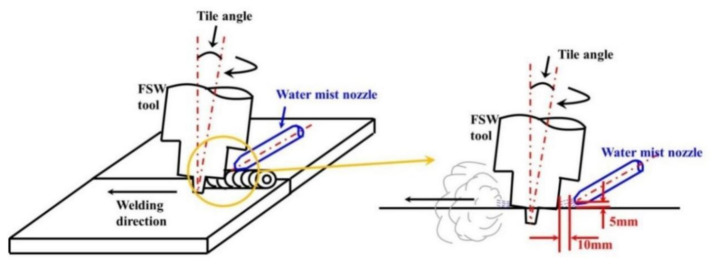
Schematic diagram of FSW with cooling medium on the surface of the workpiece [55].

**Figure 19:**
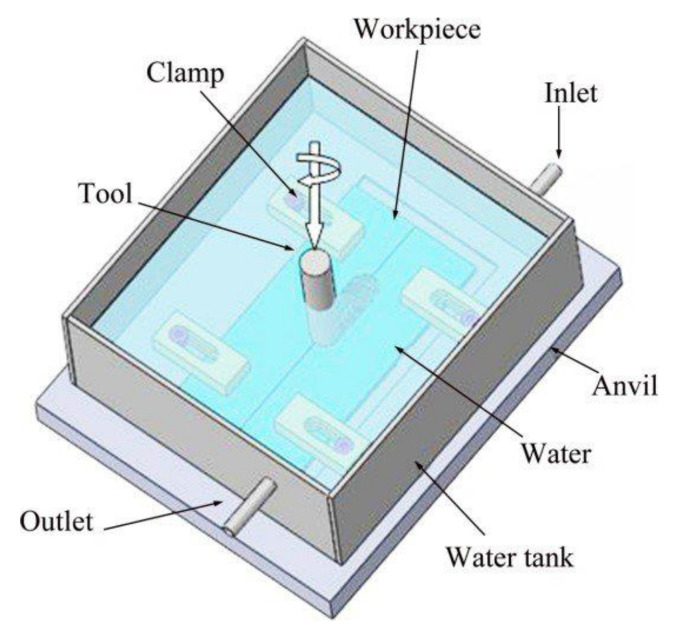
Schematic diagram of FSW with the workpiece submerged in the cooling medium [174].

According to the action position of the cooling medium, the FSW with externally assisted cooling techniques can be summarized into two categories: (I) FSW with cooling medium on the surface of the workpiece, as shown in Figure 18; (II) FSW with the workpiece submerged in the cooling medium, as shown in Figure 19.

The cooling medium has a great influence on the mechanical properties of the joint. Fratini et al. [175] carried out FSW of AA7075-T6 aluminum alloy using compressed air and water as an external cooling medium to investigate the effect of different cooling media on the mechanical performance of the joints. They reported that water provided a stronger cooling effect than compressed air and caused a stronger improvement in tensile strength. Sharma et al. [176] found the same result; namely, joints under water conditions had better tensile properties than those under compressed air conditions. However, it should not be assumed that FSW using an external cooling medium with better cooling performance can be more beneficial for the mechanical properties of the joint. This has been confirmed by the results of other researchers. Wahid et al. [130] studied joints of AA6082-T6 aluminum alloy that were produced under room-temperature water and cold water (7 °C). They found that even if cold water had a better cooling effect than room-temperature water, the size and degree of the HAZ in joints welded with cold water were also smaller, but the tensile strength of the joints under cold water conditions was lower. Fu et al. [177] investigated the FSW of AA7050-T6 aluminum alloy under cold water (8 °C) and hot water (90 °C), and they also found that the joints welded using hot water with a lower cooling effect had better mechanical properties. This “unreasonable phenomenon” is due to the fact that the strength of the joint is determined by the NZ, the TMAZ, the HAZ and the base materials. Moreover, apart from precipitate strengthening, solid solution strengthening also plays an important role in joint strength. Generally, the temperature in the NZ is greater than the solution treatment temperature of age-hardening aluminum alloys, and the precipitates can be dissolved and form a supersaturated solid solution. If the temperature in the NZ is greater than the over-aging temperature of precipitates but lower than the solution treatment temperature of age-hardening aluminum alloys, the precipitates will undergo phase transformation and coarsening, which dramatically reduces the performance of the NZ [178,179]. Hence, under some conditions, with FSW under externally assisted cooling, although the size and degree of the HAZ in the joint can be reduced, the NZ loses the solid solution strengthening effect; consequently, the tensile strength of joints under a cooling medium with better cooling performance is not higher.

### 3.2. Fusion Welding with Externally Assisted Cooling

To improve the stability of the arc and control the welding defects in the process of fusion welding of age-hardening aluminum alloys, the externally assisted cooling mode of spraying a cooling medium onto the surface of the workpiece or submerging the workpiece in the cooling medium cannot be adopted. However, a high-thermal-diffusion welding pad can reduce the heat input of the joint and improve the influence of the welding heat cycle on the performance of the welded joint. Liang et al. [180] investigated 8 mm 6N01-T5 by MIG welding with a water-cooled copper pad as an externally assisted cooling mode; they found that the amount of large-sized β′precipitates in the HAZ decreased, the softening range and degree of the joint were also reduced, and the ultimate tensile strength of the joint was 5.1% higher than under natural cooling conditions. Cao et al. [181] employed P-MIG to weld 8 mm 6N01-T5. Compared to welding under natural cooling conditions, the peak temperature decreased and the cooling rate increased during the welding process under a water-cooled copper pad, which narrowed the softening zone of the joint. The ultimate tensile strength of the joint improved by around 4.8%. However, Song et al. [182] described 1 mm 6061-T6 and 1 mm 5083-O welded by TIG with a high-thermal-diffusion water-cooled copper pad; the ultimate tensile strength of the joint was around 16.6% higher than that under natural cooling conditions. This indicates that thinner welding specimens display a more obvious improvement in joint properties during fusion welding under externally assisted cooling.

## 4. Post-Weld Treatment

Although the joint softening of age-hardening aluminum alloys can be reduced by the low-heat-input welding methods and can be controlled by the externally assisted cooling technique, softening that occurs mainly due to the precipitation strengthening effect being weakened in the FZ and HAZ is inevitable after welding. The differences in the performance of the FZ and HAZ from that of the base metal result in stress–strain concentration during stress deformation [183], which reduces the comprehensive mechanical properties of the joint. Therefore, strengthening the softening zone in joints is important to improve joint performance. In the current studies, the researchers mainly adopt post-weld heat treatment, post-weld surface modification treatment and post-weld rolling, through precipitation strengthening by the reprecipitation of the coherent strengthening phase, strain strengthening by introducing dislocation or both to improve the properties of the softening zones and entire welded joints.

### 4.1. Post-Weld Heat Treatment

Various effective measures have been developed and utilized by researchers to avoid and overcome softening when welding joints of age-hardening aluminum. However, currently, post-weld heat treatment (PWHT) is still considered the best method to improve the properties of the soft zone, especially for thick-plate joints. The main purpose of PWHT for age-hardening aluminum alloys is to cause the joints to reprecipitate the coherent precipitation phases, resulting in a homogeneous distribution of the fine strengthening precipitates and improving the mechanical properties of the joint back to the state of the base materials [56,184]. However, due to the differences in the base metals’ chemical compositions, initial temper conditions and thickness, welding methods and process parameters, the optimal heat treatment process for the joint will also be different. PWHT for joint welding by dissimilar aluminum alloys is a challenge owing to the different base metals with different optimal treatment processes, particularly for the peak-aging processes of base metals, which differ significantly. Therefore, it is not straightforward to improve the performance of dissimilar age-hardening aluminum alloy-welded joints compared to similar joints, and further study is needed [185]. PWHT is applied to the whole component and is thus limited by the dimensions [186]. In addition, with differences in joint performance regarding formability, ductility, strength, fatigue, corrosion resistance, etc., the optimal heat treatment process for the joint will also be different. Table 10 summarizes the most conventional PWHT for age-hardening aluminum alloys. It can be concluded from Table 10 that when the selected post-weld heat treatment process is better than the initial heat treatment state of the base metal, the strength of the welded joint can reach or even exceed the strength of the base metal.

### 4.2. Post-Weld Surface Modification Treatment

Stress corrosion, fatigue properties, fracture properties and other failure behaviors of welded joints are often affected by the material state near the surface [197]. Hence, post-weld surface modification treatment is one of the most important measures to improve the performance of joints. During the surface modification treatment process, severe plastic deformation occurs near the surface of the joints, which results in work hardening due to the increase in dislocation density. Moreover, some surface modification treatment processes, such as laser shot peening and cold spraying processes, not only increase the dislocation density but also result in grain refinement and precipitation of the strengthening phase, which relieves joint softening and improves the mechanical properties of joints.

#### 4.2.1. Laser Shot Peening

**Figure 20:**
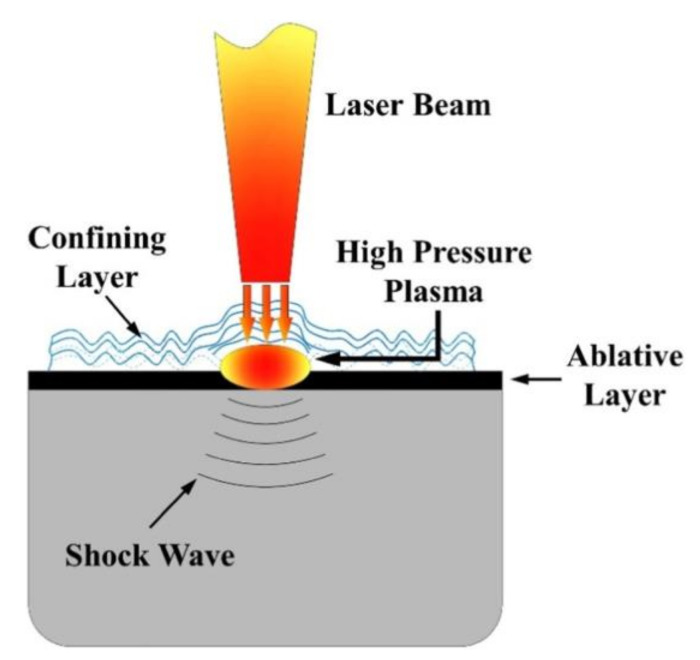
Schematic diagram of laser shock peening process [201].

High-density laser pulses (several GW/cm^2^) irradiated through a 1–2 mm confinement layer (usually water) onto an ablative layer (aluminum foil or black paint) covered by the material surface produce high-temperature and high-pressure plasma, as shown in Figure 20. The rapidly expanding plasma is bound by the confinement layer, which produces a high-pressure (GPa) pulse in a short time (ns level) and propagates into the material in the form of a shock wave [198,199,200,201]. Joint of age-hardening aluminum alloys are treated by laser shot peening, which causes a change in the surface state and microstructure under the influence of heat and force.

**Figure 21:**
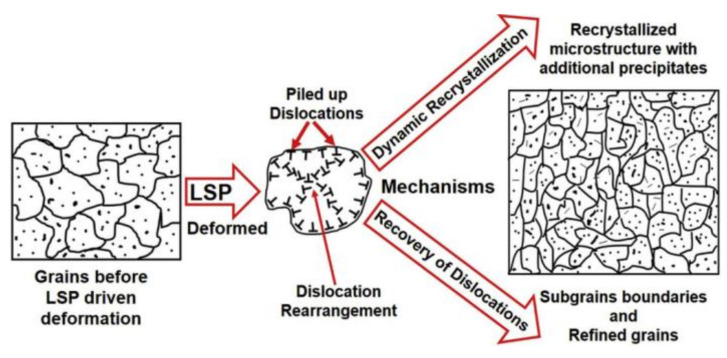
Schematic diagram of microstructure evolution during age-hardening aluminum alloy laser shot peening [202].

Dhakal and Swaroop [202] studied a 6061-T6 aluminum alloy by laser shot peening and found an increase in dislocation density and grain refinement; in addition, the high-angle grain boundary fraction was increased, and the β” phases that contributed to the strengthening effect were precipitated in the matrix, as shown in Figure 21. This resulted in improving the mechanical properties of laser shot-peened specimens. Moreover, Sun et al. [198] employed laser shock peening to treat 2 mm 6061-T6 aluminum alloy laser-welding joints and found that the porosity ratio was reduced, and the tensile strength could be increased from 203.98 MPa to 237.9 Mpa, with an increase of 14%. Hatamleh comparatively studied the performance of 12.5 mm 2195-T8 aluminum alloy FSW joints by shot peening and laser shot peening [203]. The results indicated that shot peening could only slightly improve the tensile properties of the joints. However, under the effect of high-energy laser peening, the tensile properties of the joints could be significantly improved.

#### 4.2.2. Cold Spraying

**Figure 22:**
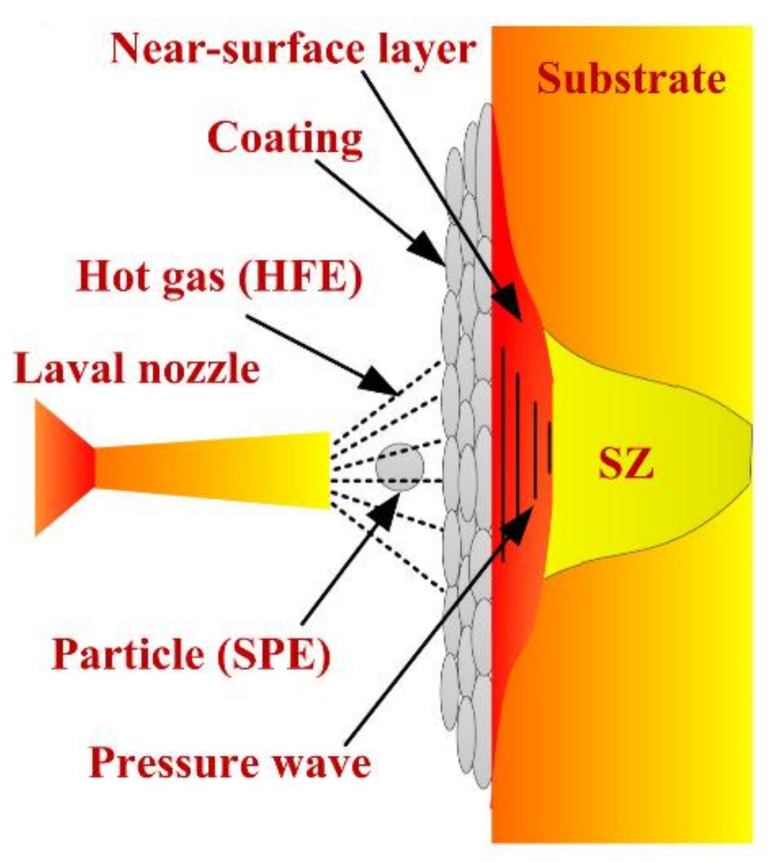
Schematic diagram of cold spraying process [57].

During the process of cold spraying, a pressurized “hot” (the temperature below the melting point of the spraying powder particles) gas passed through a Laval nozzle expands, which can accelerate the powder particles to a high velocity and violently impact the substrate, producing extensive deformations and metallurgical bonds [186,204,205], as shown in Figure 22. Under the influence of heat and force, the microstructure (grain size, state of precipitation phases and dislocation density) and performance (corrosion properties, fatigue properties and mechanical properties) of the material will change.

Li et al. [57,206,207] systematically investigated the effect of cold spraying on the performance of AA2024-T3 aluminum alloy FSW joints. The results showed that the cold spraying process could significantly improve the tensile strength, microhardness near the top surface, corrosion resistance and fatigue life of joints. The improvement was attributed to the grain refinement, the increase in the high-angle grain boundary fraction, precipitation of the strengthening phase and the reduction in residual stresses due to the effect of cold spraying on the surfaces of the joints.

### 4.3. Post-Weld Rolling Treatment

**Figure 23:**
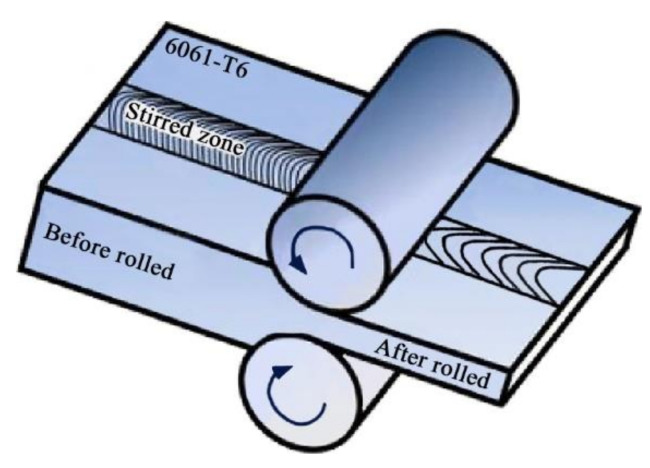
Schematic diagram of post-weld entire rolling [58].

Rolling, as an important process to increase the dislocation density and improve the strength of materials, is commonly used to strengthen welded joints. At present, the post-weld rolling treatments for strengthening age-hardening aluminum alloy joints mainly include the following two types:

(1) Post-weld entire rolling: After welding, the entire plate (including FZ, HAZ, BM) is rolled to a thickness less than the as-received base metal (the distance between the two rollers is set to less than the thickness of as-received base metal), as shown in Figure 23.

Du et al. [58] employed double-side friction stir welding of a 6 mm 6061-T6 aluminum alloy, and they post-weld rolled the entire plate to 4 mm, 3 mm and 2 mm. They found that the ultimate tensile strength of the stirring zone and BM increased as the rolling reduction increased, and the elongation of the stirring zone and BM decreased as the rolling reduction increased. The stirring zone had a weaker dislocation strengthening effect than BM. Therefore, although the weak position in the as-welded joint had been strengthened, it was still the weak position in the post-weld rolled joint. Moreover, the elongation of entire specimens would be deteriorated as the BM was strengthened by the strong strain hardening effect.

**Figure 24:**
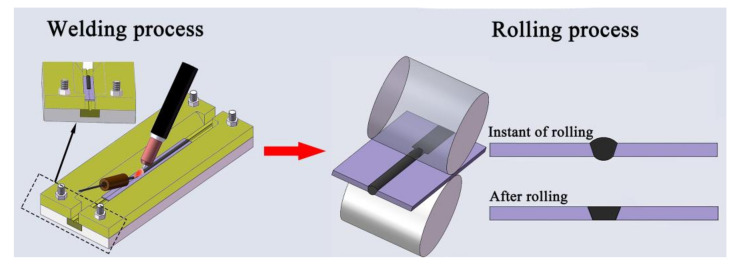
Schematic diagram of post-weld partial rolling.

(2) Post-weld partial rolling: After welding with a filler wire, only the weld reinforcement is rolled (the distance between the two rollers is set to the thickness of the received base metal), as shown in Figure 24.

Song et al. [182] found that the softening of joints of age-hardening aluminum alloys presented a certain gradient characteristic, so they proposed a new joining technique of welding with a filler wire under the water−cooled copper pad cooling conditions and subsequent rolling of the weld reinforcement. The joining technique combined the externally assisted cooling technique and the post-weld partial rolling process. It reduced the softening range and softening degree of joints by the externally assisted cooling technique, and caused the joints to produce a gradient dislocation density by the post-weld partial rolling process. Compared to the welding specimen, the tensile strength of the new joining specimen had an increase of 46.5%. Because the BM had not been strengthened by the strong strain hardening effect, the fracture elongation of the new joining specimen had an increase of 50.5%.

## 5. Discussion

The over-aging phenomenon of the strengthening phase occurs during the welding process of age-hardening aluminum alloy, which seriously reduces the mechanical properties of the welded joint. The softening range and degree of strengthening phase in the joint are closely related to the welding heat input. Compared with traditional TIG, MIG and other large-heat-input welding heat sources, LBW, FSW and CMT, with low heat input, have more significant advantages in the welding of age-hardening aluminum alloys, as they can greatly reduce the softening degree and range of welded joints and improve the performance of joints. In addition, welding researchers also found that the softening of the joint is related not only to the peak temperature range during welding, but also to the residence time at high temperatures. Therefore, the externally assisted cooling technique is adopted in the welding process to reduce the peak temperature range and increase the cooling rate, so as to control the influence of the welding thermal cycle on the strengthening phase. In most cases, the mechanical properties of the joint can be further improved. Although the low-heat-input welding method and externally assisted cooling process improve the joint softening of age-hardening aluminum alloys, the softening of the weld seam and heat-affected zone remain and often represent a weakness in the service process. Therefore, strengthening the post-weld joint softening zone is of great significance to improve the joint performance. In current studies, researchers mainly adopt post-weld heat treatment, post-weld surface modification treatment and post-weld rolling, through precipitation strengthening by the reprecipitation of the coherent strengthening phase, strain strengthening by introducing dislocation or both to improve the properties of the softening zones. However, currently, PWHT is still considered the best method to improve the properties of the soft zone, especially for thick-plate joints. However, due to the differences in the base metal chemical compositions, initial temper conditions and thickness, welding methods and process parameters, the optimal heat treatment process of the joint will also be different. PWHT for joints welded by dissimilar aluminum alloys is a challenge, and research is ongoing. This is due to the different base metals requiring different optimal treatment processes, particularly for the peak-aging processes of base metals, which differ significantly.

## 6. Conclusions

Age-hardening aluminum alloy is a kind of non-ferrous metal that can meet the worldwide requirements of energy-saving, emission reduction and light weight. However, the precipitation strengthening effect is easily affected by the welding heat input. To improve the performance of joints welded by conventional fusion welding, which results in a large welding softening range and severe softening degree, researchers have adopted many effective welding processes and methods, such as low-heat-input welding methods, externally assisted cooling techniques and post-weld treatment techniques. Although studies have made great achievements, the joint performance remains lower than that of the base metal and there is room for improvement. In addition, PWHT for dissimilar age-hardening aluminum alloys is a challenge owing to the different base metals requiring different optimal treatment processes. Therefore, it is necessary to carry out further study as follows:

(1) Further research on the softening mechanism of aluminum alloys should be carried out to establish the relationship between the welding thermal cycle and joint grain size, strengthening phase characteristics, dislocation density and texture, in order to transform the analysis of the effect of welding heat input on joint softening and performance from qualitative analysis to quantitative analysis.
(2) It is necessary to establish the precise response mechanism of an over-aged microstructure and the temperature field of a welded joint, monitor the state of the welding heat source and the information about the plate temperature field in real time and dynamically and scientifically control the parameters of the welding heat source, in order to precisely control the shape and performance of joints during the welding process.
(3) According to the softening characteristics of the joint, it is necessary to develop post-weld local strengthening treatments to achieve the cooperative strengthening and toughening of the properties of each zone of the welded joint.
(4) It is necessary to establish the relationship between the softening characteristics of the joint and fatigue performance, corrosion performance and forming performance, to promote the research and development of age-hardening aluminum alloy tailor-welded blank manufacturing technology and to expand the scope of application of the materials.
(5) It is necessary to research the evolution of the microstructure and properties in the softening zone of welded joints and the strengthening of joints over time, to provide theoretical and data support for evaluating the service performance of structural parts.

## Figures and Tables

**Table 1:** The classification of heat-treatable aluminum alloys and non-heat-treatable alloys. Adapted from [7,12]. (Guinier-Preston zone (GP)).

Series	Main Alloying Elements	Type	Precipitate Sequence
1XXX	Pure Al (99% or greater)	Non-heat-treatable	
2XXX	Al-Cu alloys	Heat-treatable	GP→θ″(coherent precipitate)→ θ′(semi-coherent precipitate)→θ(CuAl_2_, non-coherent precipitate)
3XXX	Al-Mn alloys	Non-heat-treatable	
4XXX	Al-Si alloys	Non-heat-treatable	
5XXX	Al-Mg alloys	Non-heat-treatable	
6XXX	Al-Mg-Si alloys	Heat-treatable	GP→β″(coherent precipitate)→ β′(semi-coherent precipitate)→β(Mg_2_Si, non-coherent precipitate)
7XXX	Al-Zn-Mg-Cu alloys	Heat-treatable	GP→η″(coherent precipitate)→ η′(semi-coherent precipitate)→η(MgZn_2_, non-coherent precipitate)
8XXX	Al + other elements	Al-Li Heat-treatable	δ′(Al_3_Li, coherent precipitate)→ δ (AlLi, non-coherent precipitate)

**Table 2:** Basic temper designation of aluminum alloys. Adapted from [10,11].

Basic Temper	Definition
F (As fabricated)	During the shaping process, the thermal condition or strain hardening does not need any special control.
O (Annealed)	Aluminum alloys are treated under high-temperature conditions to obtain optimal toughness, ductility and workability.
H (Strain hardened)	Aluminum alloys are treated by strain-hardening method to increase the strength, with or without supplementary thermal treatment.
W (Solution heat-treated)	An unstable temper, rather limited designation. The aluminum alloys are subjected to spontaneous aging at room temperature after solution heat treatment.
T (Thermally treated)	Aluminum alloys are thermally treated to produce stable tempers different to F, O or H, with or without supplementary strain-hardening.

**Table 3:** Subdivision of T temper of aluminum alloys. Adapted from [10].

Subdivision of T Temper	Process
T1	Cooled from a high-temperature shaping process and naturally aged.
T2	Cooled from a high-temperature shaping process, cold worked, and naturally aged.
T3	Solution heat-treated, cold worked, and naturally aged.
T4	Solution heat-treated and naturally aged.
T5	Cooled from a high-temperature shaping process and artificially aged.
T6	Solution heat-treated and artificially aged.
T7	Solution heat-treated and over-aged/stabilized.
T8	Solution heat-treated, cold worked, and artificially aged.
T9	Solution heat-treated, artificially aged, and cold worked.
T10	Cooled from a high-temperature shaping process, cold worked, and artificially aged.

**Table 5:** Summary of the tensile properties of autogenous LBW age-hardening aluminum alloy butt joint.

BM	Temper	BM Thickness (mm)	Ultimate Strength (MPa)		Joint Efficiency = Joint/BM (%)	Fracture Location	Ref.
			BM	Joint			
2024	T3	3	463.0	364.0	78.6	FZ	[62]
2060	T8	2	494.4	315.0	63.7	FZ	[63]
2060	T8	1	497.5	347.7	69.9	FZ	[63]
2090	T8	1.6	408.0	335.0	82.1	FZ	[64]
6022	T4	1	232.9	170.0	73.0	FZ	[65]
6061	T4	1	250.0	230.0	92.0	FZ	[66]
6061	T6	4	−	220.0~231.0	~70.0	FZ	[67]
7075	T6	2	592.1	358.2	60.5	FZ	[68]
7075	T6	2	588	406.0	69.0	FZ	[69]
Al-Zn-Mg-Cu	T6	2	675.9	471.1	69.7	FZ	[70]

**Table 6:** Comparison of the tensile properties of the age-hardening aluminum alloy butt joint welded by conventional MIG and laser–MIG.

BM/ Temper	Plate Thickness (mm)	Ultimate Strengthof BM (MPa)	Filler Wire	Welding Method	Ultimate Strength of Joint (MPa)	Joint Efficiency= Joint/BM (%)	Ref.
2519 T87	20	479	Self-made	MIG	275	57.4	[94]
				Laser-MIG	295	61.6	
6005 T6	5	276	ER5356	MIG	190	68.8	[95]
				Laser-MIG	206	74.6	
6N01S T5	8	292	ER5356	MIG	210	71.9	[96]
				Laser-MIG	243	83.2	
6005A T6	4	303	ER5087	MIG	225	74.3	[97]
				Laser-MIG	250	82.5	
6N01 T5	4	260	ER5356	MIG	183	70.4	[98]
				Laser-MIG	202	77.7	

**Table 7:** Summary of the tensile properties of a conventional FSW age-hardening aluminum alloy butt joint (nugget zone (NZ), thermo-mechanically affected zone (TMAZ)).

BM	Temper	BM Thickness (mm)	Ultimate Strength (MPa)		Joint Efficiency = Joint/BM (%)	Fracture Location	Ref.
			BM	Joint			
2014	T6	1	329.0	242.0	73.6	TMAZ	[113]
2014	T6	5	470.0	285.0	60.6	interface of NZ and TMAZ	[114]
2014	T651	6	469.0	323.0	68.9	HAZ	[13]
2017	T451	6	446.0	351.03	78.7	NZ	[115]
2024	T351	6	481.0	409.0	85.0	interface of NZ and TMAZ	[116]
2024	T351	6	481.0	395.0	82.1	interface of NZ and TMAZ	[117]
2060	T8	2	530.0	440.0	83.0	NZ	[118]
2060	T8	2	532.0	435.0	81.0	interface of NZ and TMAZ	[119]
2198	T8	3.2	488.5	397.2	81.3	NZ	[120]
2219	T6	13	443.0	298.0	67.3	TMAZ	[121]
6005	T6	10	285.0	215.0	75.4	HAZ	[22]
6061	T4	6.35	−	229.0	93.0	HAZ	[122]
6061	T6	0.8	351.7	292.6	83.2	HAZ	[123]
6061	T6	1	304.0	265.0	87.2	interface of HAZ and BM	[124]
6061	T6	3	322.0	233.0	72.4	NZ	[125]
6061	T6	5	270.0	202.0	74.8	interface of HAZ and TMAZ	[126]
6061	T6	6	283.0	246.0	86.9	HAZ	[127]
6082	T6	2	310.0	202.67	65.4	HAZ	[128]
6082	T6	2	318.4	218.5	68.6	HAZ	[129]
6082	T6	3	305.0	216.0	70.8	HAZ	[130]
7020	T6	4.5	372.0	353.0	95.0	NZ	[131]
7039	T6	5	414.0	354.4	85.6	HAZ	[132]
7055	T4	1.8	542.5	450.2	83.0	TMAZ	[133]
7075	T6	0.5	544.6	482.0	88.5	interface of NZ and TMAZ	[134]
7075	T6	1.5	585.0	500.0	85.5	TMAZ	[135]
7075	T6	5	586.0	487.0	83.1	HAZ	[136]
7075	T651	6.35	622.0	468.0	75.2	HAZ	[137]

**Table 8:** Summary of the tensile properties of age-hardening aluminum alloy butt joint welded by conventional CMT welding.

BM	Temper	BM Thickness (mm)	Filler Wire	Ultimate Strength (MPa)		Joint Efficiency = Joint/BM (%)	Fracture Location	Ref.
				BM	Joint			
2198	T8	2	ER4043	470.0	270.0	57.4	Fusion line	[160]
6005	T6	3	ER4043	270.0	171.6	63.6	FZ	[159]
6061	T6	3	ER4043	318.0	215.0	67.6	HAZ	[161]
6082	T4	2	ER5356	296.0	−	79.0	FZ	[162]
7075	T6	2	ER5356	−	−	60.0	FZ	[154]
7075	T6	2	ER5356	597.0	312.0	52.3	FZ	[163]
7475	T761	2.38	ER5356	592.0	531.0	89.7	FZ	[153]
7475	T761	2.38	ER4043	592.0	452.0	76.4	FZ	[153]

**Table 9:** Comparison of the tensile properties of age-hardening aluminum alloy butt joint welded by FSW under normal and externally assisted cooling conditions.

BM	Temper	BM Thickness (mm)	Ultimate Strengthof BM (MPa)	Cooling Condition	Ultimate Strength of Joint (MPa)	Joint Efficiency = Joint/BM (%)	Ref.
2014	T651	6	469	normal	323	68.9	[13]
				water cooling	379	80.8	
2219	T6	7.5	432	normal	340	78.7	[168]
				underwater	360	83.3	
2519	T87	6	452	normal	267	59.1	[169]
				underwater	345	76.3	
6061	T6	3	310	normal	239	77.1	[170]
				underwater	274	88.4	
6061	T6	6	−	normal	182	−	[171]
				underwater	218	−	
6082	T6	3	305	normal	215	70.5	[172]
				underwater	241	79.0	
7075	T6	−	−	normal	−	~70.5	[173]
				underwater	−	~73.5	

**Table 10:** The mechanical properties of joints subjected to post-weld heat treatment in the literature (ST: solution treatment, AT: aging treatment).

Materials /Ref.	BM Thickness	Welding Process	Ultimate Strength (MPa)		As-Welded Joint Efficiency	PWHT Process	PWHTed Joint Efficiency	Conclusion
			BM	Joint				
2219-T87 [187]	8 mm	Variable polarity tungsten inert gas (VPTIG) welding with ER2325 filler wire	470.0	250.0	53.2%	ST:535 °C, 30 min AT:175 °C, 12 h	76.0%	The microstructure of the PWHTed joint is more homogeneous than that of the as-welded joint; PWHTed joints not only have better tensile strength but also have better stress corrosion cracking resistance.
2219-T6 [188]	8 mm	(1) VPTIG welding with ER2325 filler wire	425.0	(1) 246.0 (2) 242.0	(1) 57.9% (2) 56.9%	AT:175 °C, 12 h	(1) 63.8% (2) 69.6%	After post-weld aging treatment, more strengthening phases reprecipitate in HAZ of pre-weld solution-treated joint.
		(2) ST: 535 °C, 1.5 h VPTIG welding with ER2325 filler wire						
6061-T6 2024-T6 [189]	4 mm	FSW(1) Advancing Side: AA6061 Retreating Side: AA2024	310.0 492.0	(1)183.0 (2) 184.0	(1) 59.0% (2) 59.0%	ST: 520 °C, 1 h AT: 165 °C, 18 h	(1) 82.5% (2) 74.0%	The position of alloys does not affect the strength of the joint, but it influences the strength of the joint by PWHT.
		(2) Advancing Side: AA2024 Retreating Side: AA6061						
2024-T3 [190]	6 mm	FSW	412.0	309.0	75.0%	(1) ST: 493 °C, 2 h AT: 190 °C, 10 h (2) ST: 493 °C, 2.5 h AT: 200 °C, 10 h	(1) 94.2% (2) 92.7%	The evolution of strengthening phases affects not only strength but also ductility and fatigue crack growth rates.
Al-Cu-Li T8 (solution treated at 540 °C for 1 h, 5% deformation, aged at 152 °C for 5 h) [191]	2 mm	FSW	413.0	379.0	91.8%	Deformation: 3% rolling AT: 152 °C, 30 h	124.7%	The pre-deformation before aging treatment introduces a large number of additional dislocations into the joint; the high density of dislocations usually act as nucleation sites for strengthening precipitates.
7075-T6 [192]	3.17 mm	FSW: (1) rotation rate: 1000 rpm Travel speed: 150 mm/min	563.5	(1) 449.9 (2) 382.1	(1) 79.8% (2) 67.8%	ST: 485 °C, 4 h AT:140 °C, 6 h	(1) 89.1% (2) 90.8%	The joints have different tensile properties, mainly owing to the different welding process parameters. However, after the post-weld heat treatment, the joints have similar tensile strength.
		(2) rotation rate: 1500 rpm Travel speed: 400 mm/min						
6013-T4 [193]	1.6 mm	(1) LBW with AlSi12 alloy powder	345.0	(1) 316.0 (2) 297.0 (3) 285.0 (4) 250.0 (5) 291.0 (6) 247.0	(1) 91.6% (2) 86.1% (3) 82.6% (4) 72.5% (5) 84.3% (6) 71.6%	AT:191 °C, 4 h	(1) 104.9% (2) 100.0% (3) 99.1% (4) 76.8% (5) 99.4% (6) 78.0%	The chemical composition and type of filler powder can not only influence the performance of as-welded joints, but also have strong effect on the performance of PWHTed joints.
		(2) LBW with AlSi12 mixed powder						
		(3) LBW with AlSi12Mg5 alloy powder						
		(4) LBW with AlSi12Mg5 mixed powder						
		(5) LBW with AlMgSi1 alloy powder						
		(6) LBW with AlMgSi1 mixed powder						
6061-T6 7075-T6 [194]		Friction welding	304.0 569.0	228.0	75.0%	(1) ST: 500 °C, 1 h (2) ST:520 °C, 1 h AT: 160 °C, 8 h (3) AT: 160 °C, 8 h	(1) 67.1% (2) 96.0% (3) 84.2%	Solution-treated (1) joint has lower tensile strength due to the complete dissolution of finer precipitates; Artificially aged (3) joint has marginally improved tensile strength due to the formation of finer precipitates; Solution+ artificially aged (2) joint has dramatically improved tensile strength due to the formation of many finer precipitates.
7039-T6 [195]	5 mm	FSW	414.0	354.4	85.6%	(1) AT: room temperature, more than one year (2) AT: 120 °C, 18 h (3) AT: 100 °C, 8 h +150 °C, 24 h (4) ST: 480 °C, 0.5 h (5) ST: 480 °C, 1 h AT: 165 °C, 6 h	(1) 94.9% (2) 92.1% (3) 74.6% (4) 61.3% (5) 73.7%	Natural aging (1) results in the formation of fine and uniformly distributed strengthening precipitates in joint compared to other PWHT; artificial aging (2) and step aging (3) result in the joint forming agglomerated spherical strengthening precipitates; solution treatment (4) and solution+ artificial aging (5) result in strengthening precipitates being dissolved in weld nugget zone and severely coarsened in HAZs.
2024-T4 [196]	3 mm	FSW	492.0	389.0	79.0%	(1) ST: 510 °C, 2.5 h AT: room temperature, 8 months (2) ST: 510 °C, 2.5 h AT:100 °C, 10 h (3) ST: 510 °C, 2.5 h AT:190 °C, 10 h	(1) 81.7% (2) 77.0% (3) 87.4%	PWHT can improve the FSW joint efficiency, but solution heat treatments at temperatures above 500 °C can cause abnormal coarsening of the grains in the weld zone, which results in mechanical properties lower than the base metal.
7075-T6 [184]	5 mm	FSW	559.0	460.0	82.3%	(1) ST: 400 °C, 0.25 h + 450 °C, 0.25 h + 400 °C, 0.25 h + 450 °C, 0.25 h + 400 °C, 0.25 h + 450 °C, 0.25 h (frequently heated between 400 °C and 480 °C for 1.5 h) (2) ST: (1) AT: 130 °C, 6 h (3) ST: (1) AT: 130 °C, 12 h (4) ST: (1) AT: 130 °C, 18 h (5) ST: (1) AT: 130 °C, 24 h (6) ST: (1) AT: 130 °C, 36 h	(1) 88.1% (2) 94.7% (3) 97.3% (4) 100.0% (5) 110.8% (6) 89.1%	The cyclic solution treatment results in the repetitive partial dissolution of large precipitates, contributing to formation of fine, metastable precipitates, and abnormal grain growth phenomenon does not take place. The peak-aged state is 130 °C, 24 h. Aging for 36 h causes precipitate over-aging; the coarsening and agglomeration of precipitate particles occurs.

## Data Availability

Data sharing does not apply to this article as no new data were created or analyzed in this study.
